# Corruption Kills: Estimating the Global Impact of Corruption on Children Deaths

**DOI:** 10.1371/journal.pone.0026990

**Published:** 2011-11-02

**Authors:** Matthieu Hanf, Astrid Van-Melle, Florence Fraisse, Amaury Roger, Bernard Carme, Mathieu Nacher

**Affiliations:** 1 Centre d'Investigation Clinique Epidémiologie Clinique Antilles Guyane CIC-EC INSERM CIE 802, Cayenne General Hospital, Cayenne, French Guiana; 2 Team EA 3593, Department of Parasitology and Mycology, Université des Antilles et de la Guyane, Cayenne, French Guiana; Burnet Institute, Australia

## Abstract

**Background:**

Information on the global risk factors of children mortality is crucial to guide global efforts to improve survival. Corruption has been previously shown to significantly impact on child mortality. However no recent quantification of its current impact is available.

**Methods:**

The impact of corruption was assessed through crude Pearson's correlation, univariate and multivariate linear models coupling national under-five mortality rates in 2008 to the national “perceived level of corruption” (CPI) and a large set of adjustment variables measured during the same period.

**Findings:**

The final multivariable model (adjusted R^2^ = 0.89) included the following significant variables: percentage of people with improved sanitation (p.value<0.001), logarithm of total health expenditure (p.value = 0.006), Corruption Perception Index (p.value<0.001), presence of an arid climate on the national territory (p = 0.006), and the dependency ratio (p.value<0.001). A decrease in CPI of one point (i.e. a more important perceived corruption) was associated with an increase in the log of national under-five mortality rate of 0.0644. According to this result, it could be roughly hypothesized that more than 140000 annual children deaths could be indirectly attributed to corruption.

**Interpretations:**

Global response to children mortality must involve a necessary increase in funds available to develop water and sanitation access and purchase new methods for prevention, management, and treatment of major diseases drawing the global pattern of children deaths. However without paying regard to the anti-corruption mechanisms needed to ensure their proper use, it will also provide further opportunity for corruption. Policies and interventions supported by governments and donors must integrate initiatives that recognise how they are inter-related.

## Introduction

Corruption is a complex problem which threatens the impact of public investments, health care access and services, equity and outcomes [1], [2]. Increasingly, health sector leaders, and citizens of all countries, are recognizing the pernicious effects of corruption both at micro and macro levels, and the need to take action [1], [3], [4]. At the level of individuals and households, there is mounting evidence of the negative effects of corruption on the health and welfare of citizens [5], [6]. The 2006 report of transparency International asserts that the corruption level undermines the achievement of the Millennium Development Goal (MDGs) and that the corruption is one of the primary causes of the fact that the global community is already off target to meet the MDGs [1]. Based on literature and previous works, Vian et al. [2] distinguished 7 areas in the health sector where corruption could have pejorative health consequences: construction and rehabilitations of health facilities, purchase of equipment and supplies, distribution and use of drugs, regulation of qualities in products, education of health professionals, medical research, and provision of services by medical personnel.

Health impacts of corruption can not be only summarized as those acting directly on health access and services. For example, nearly 1.2 billion people in the world do not have guaranteed access to water and more than 2.6 billion are without adequate sanitation, with devastating consequences for development and poverty reduction. In developing countries, about 80 per cent of health problems can be linked back to inadequate water and sanitation [7]. The human consequences of this water crisis are devastating and affect the poor and women most of all. Experts concur that the water crisis is a crisis of water governance where, as suggested elsewhere, corruption could be one of the main cause and catalyst for this crisis and that it could affect all aspects of the water and sanitation sector, from resources management to services [8]. More globally corruption was shown to affect all domains of life from education to economic performances [4] and thought these ways could also impact health outcomes.

**Figure 1:**
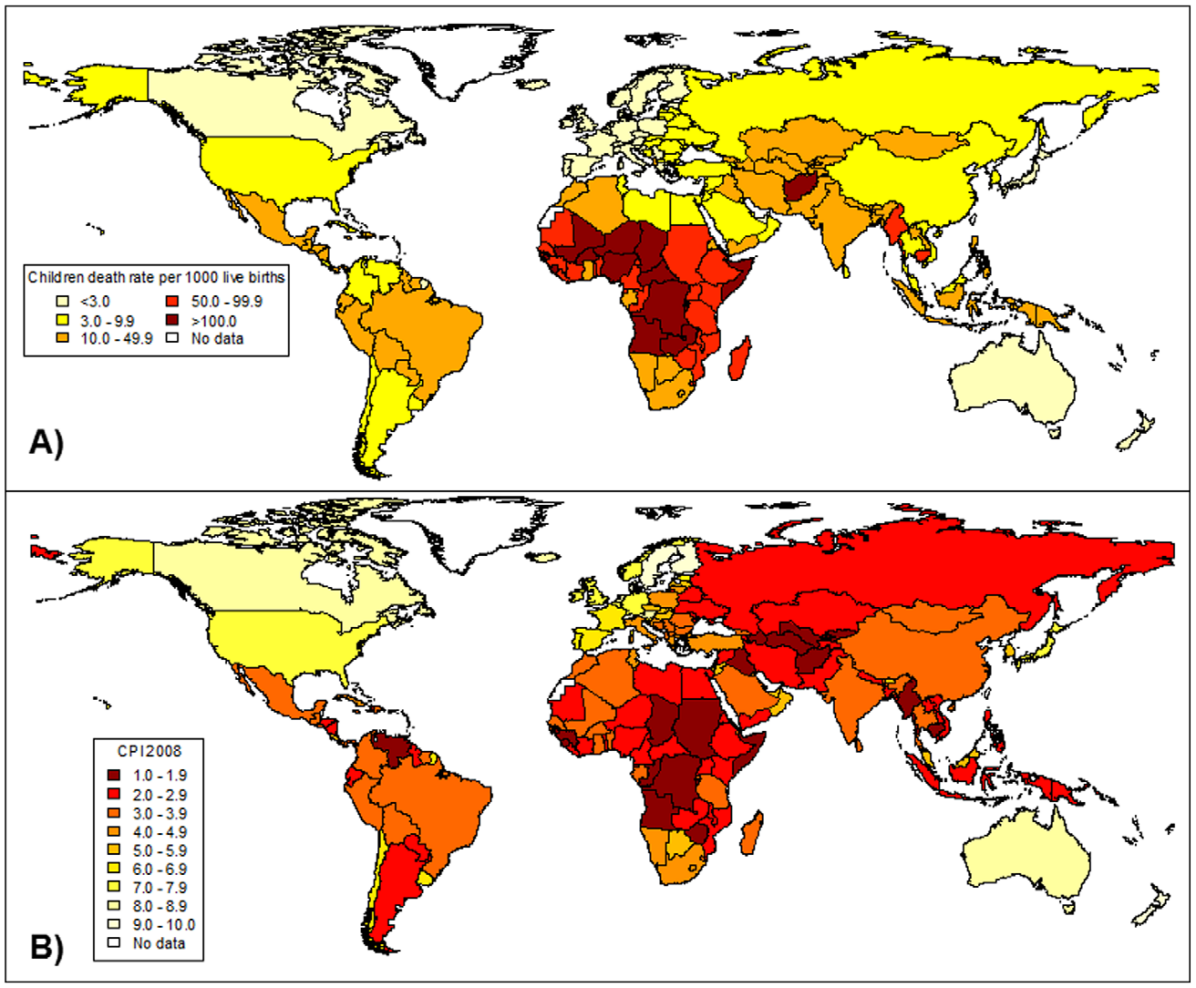
World repartition of children deaths and perceived corruption. A) Deaths of children younger than 5 years per 1000 live births in 2008. Derivated from data compiled by Black and colleagues [11]. B) Corruption Perceptions Index (CPI) in 2008. Derivated from data compiled by Transparency International [10].

Among major health problems, child mortality is one of the most preoccupying. In 2006, for the first time since records have been kept, the number of children dying before their fifth birthday fell below 10 million, to 9.7 million. In 2008, it was estimated that 8.795 million deaths under 5 years of age occurred worldwide (figure 1). This milestone follows a long-term decline in the global under-five mortality rate since 1960. However, many countries still have high levels of child mortality, particularly in sub-Saharan Africa and South Asia, and in recent years have made little or no progress in reducing the number of child deaths. This global progress is insufficient to achieve MDGs in which reduction of mortality is crucial. The United Nations aim to decrease the child mortality rate by two-thirds between 1990 and 2015 [9], [10]. By its influences on health, water/sanitation services, education and economic development, corruption could potentially have a great impact on the dynamics of child mortality. However quantitative data on this subject at a population scale are poor. To our knowledge, the most recent study quantifying the specific link between these two variables was the one realised by Abed and colleagues [3]. This study, in 2002, observed a significant link between corruption level and child mortality but only incorporated a limited set of adjustment variables and was made using 10 years old data. A more recent vision of current global impacts of corruption on child mortality is needed to implement efficient programs and improve children survival.

In this perspective, data on national under five mortality rates, the classical risk factors of child mortality, and corruption level were gathered circa 2008. By using linear regressions, this ecological study aimed to 1) assess at a country scale the current impacts of corruption on global children mortality rate after adjusting with a large set of variables, and 2) roughly calculate the number of children deaths attributable to corruption.

## Materials and Methods

### Data source

In this population based study, national data on under five mortality presented as the number of deaths per 1000 live births in 2008 were compiled from estimations calculated by Black and colleagues [11] and freely available on the Gapminder project website [12]. National corruption level was assessed thought the 2008 CPI compiled by Transparency International which orders the countries of the world according to “the degree to which corruption is perceived to exist among public officials and politicians” [13]. This index is ranging from 0 to 10 with a higher score meaning less (perceived) corruption.

According to the literature, the main risk factors of children deaths are access to drinkable water and sanitation facilities [9], [14], [15], socio-economic conditions [9], [16], [17], vaccination coverage [18], health services level [9], [19], undernutrition [20], [21], climate [22], [23], war and natural disasters [24], and political context and corruption [1], [3]. Adjustment variables were categorized into four groups:

#### Socio economic factors

To reflect the economic and development level of studied countries, the Gross Domestic Product (GDP) per capita in 2008 and the dependency ratio (percentage of working-age population) in 2008 were used [25].

National level of water and sanitation facilities were assessed thought the two following World Bank indicators [25]: the 2008 percentage of the population with at least adequate access to excreta disposal facilities and 2008 percentage of the population with reasonable access to an adequate amount of water from an improved source.

Education level was assessed by the literacy rate in 2008 which was compiled from 2 different sources of data [25], [26].

Two major population characteristics obtained from the World Bank [25] were also used: the percentage of rural population in 2008 and the population density per square km in 2008.

#### Health and medical factors

The national health services level was assessed thought the total health expenditure per capita in 2008 and thought the percentage of GDP invested in health expenditure [25].

Vaccination coverage was assessed thought the percentage of children ages 12–23 months immunised against DTP and measles [25].

The nutritional level was measured by the 2007 food supply (kilocalories/person/day) compiled by the FAO [27].

#### Environmental factors

National climates were categorized according to the revised Köppen-Geiger climate classification [28]. Four derived variables were created: presence on the national territory of an equatorial climate, an arid climate, a warm temperate climate and a snow or polar climate.

Impacts of floods were measured through the percentage of national population affected by flood compiled by the Center for Research on the Epidemiology of Disasters (CRED) [29].

#### Political and societal factors

Impact of war on children deaths was measured thought the national battle-related deaths/person derivated from World Bank indicators [25].

Democracy was measured thought two perceived scores on civil liberties and political rights calculated by freedom house, an international non-governmental organization that conducts research and advocacy on democracy, political freedom and human rights [30]. These indexes are ranging from 1 to 7 with a higher score meaning less (perceived) civil liberties and political rights.

### Statistical analysis

Logarithm transformations of national data on deaths of children younger than 5 years per 1000 live births in 2008 were used in this study as the outcome variable. Data on 178 countries were provided.

To understand the crude correlation structure between all the quantitative variables incorporated in the model (dependent and independent variables), a Focused Principal Components Analysis was performed [31]. Compared to a traditional crude correlation matrix, the FPCA gives a multidimensional graphical display allowing, in one look, to simply understand the correlation structure between the outcome and exploratory variables and between exploratory variables themselves. The representation obtained with this method is close to a Principal Components Analysis (PCA). Contrary to PCA, correlations (Pearson's correlation) between the dependent variable and the other variables are represented faithfully. The relationships between non dependent variables are interpreted like in a PCA: correlated variables are close or diametrically opposite (for negative correlations), independent variables make a right angle with the origin. The package “psy” of the R statistical language was used to perform the Focused Principal Components Analysis.

Unweighted univariate linear regressions were then performed. In view to identify the dominant factors in those that were statistically significant in univariate regression (p<0.05), significant variables were included in a multivariate linear regression model. To determine the final multivariate model, a backward stepwise linear regression was then used to simultaneously adjust for various variables of interest. Statistical significance was set at p<0.1.

As a step towards stabilizing the residual variation in regression analysis, logarithmic transformations of the data on health expenditure per capita, GDP per capita, battle-related deaths/person, percentage of national population affected by floods were used. Homoscedasticity was verified graphically and the model including corruption was compared to the model without corruption using the adjusted R^2^. Multicollinearity and variance inflation factors were also checked in the final linear model to assess its robustness. All statistical analyses were conducted using R 2.12.0 [32].

## Results

National data on under five years deaths per 1000 live births and CPI in 2008 are shown in figure 1. Variables that can potentially explain inter-country variation in the child mortality rate were summarized in table 1. In 2008, the child mortality rate varied from 1.027 (Slovenia) to 195.188 (Afghanistan) per 1000 live births with a world median at 11.789.

**Table 1:** Available variables that can potentially explain inter-country variation in the rate of deaths in children in 178 countries, 2008.

	No. of countries with data (%)	Value range	Reference
**Socio economic factors**			
*GDP per capita (2008)*	171 (96%)	144.8–117954.7	[25]
*People with access to improved water source (2008) (%)*	172 (97%)	45–100	[25]
*People with access to improved sanitation (2008) (%)*	161 (90%)	9–100	[25]
*Percentage of rural population (2008) (%)*	178 (100%)	0–89.6	[25]
*Literacy rate (circa 2008) (%)*	167 (94%)	27.6–100	derivated from [25] and [26]
*Dependancy ratio (2008)*	178 (100%)	20.89–106.95	[25]
*Density per square km (2008)*	178 (100%)	1.7–6757.0	[25]
**Health and medical factors**			
*Total health expenditure per capita (2008)*	171 (96%)	10.3–8071.1	[25]
*Health expenditure (2008) (% GDP)*	175 (98%)	1.949–15.180	[25]
*DTP coverage (2008) (%)*	178 (100%)	23–99	[25]
*Measles coverage (2008) (%)*	178 (100%)	23–99	[25]
*Food supply (2007) (kilocalories/person/day)*	166 (93%)	1605–3819	[27]
**Environmental factors**			
*Presence of an equatorial climate on the national territory*	178 (100%)	-	derivated from [28]
*Presence of an arid climate on the national territory*	178 (100%)	-	derivated from [28]
*Presence of a warm temperate climate on the national territory*	178 (100%)	-	derivated from [28]
*Presence of a polar or snow climate on the national territory*	178 (100%)	-	derivated from [28]
*National population affected by flood (2008) (%)*	178 (100%)	0–13.1	Derived from [29]
**Political and societal factors**			
*Civil liberties (2008)*	177 (99%)	1–7	[30]
*Political rights (2008)*	177 (99%)	1–7	[30]
*Corruption perception index (2008)*	168 (94%)	1.0–9.3	[13]
*National battle-related deaths/person (2008)*	178 (100%)	0.00–0.05	[25]

**Figure 2:**
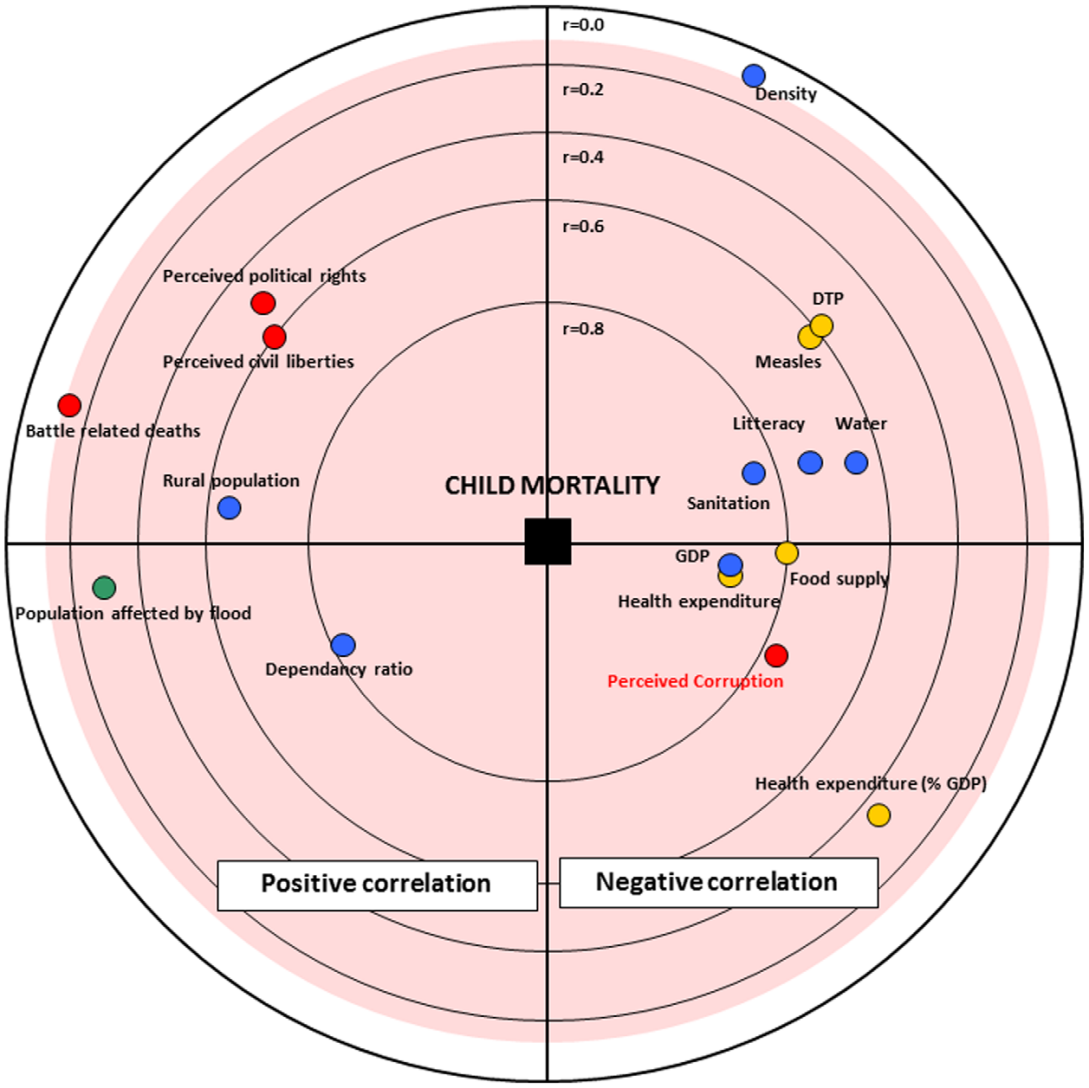
Pearson's correlations between the mortality rate in children and the other variables used in the analysis. Representation obtained with a Focused Principal Components Analysis. Pearson's correlations between the mortality rate in children and the other variables are represented faithfully. Positive correlations with child mortality are plotted on the left and negative ones on the right. Correlations which are significantly different to zero are inside the pink circle. The relationships between the other variables are interpreted like in a PCA: correlated variables are close or diametrically opposite (for negative correlations), independent variables make a right angle with the origin. Colours indicate the nature of explicative variables (red: political and societal factors, blue: socio economic factors, orange: health and medical factors and green: environmental factors).

A synthesis of Pearson's correlations between all quantitative variables used in this analysis was presented in figure 2 (obtained with a Focused Principal Components Analysis). The six variables the most correlated with log of mortality rate in children were: the log of health expenditure per capita (r = −0.87), the log of GDP per capita (r = −0.86), the percentage of people with access to improve sanitation (r = −0.84), the dependency ratio (r = 0.81), the food supply (r = −0.78) and the perceived corruption (r = −0.76). The six variables the most correlated with the perceived corruption index were: the health expenditure per capita (r = 0.82), the GDP per capita (r = 0.78), civil liberties index (r = −0.69), the food supply (r = 0.66), the political rights index (r = −0.64) and percentage of rural people (r = −0.58).

In the univariate analysis, all predictor variables (excepted battle-related deaths/person) were significantly associated (p<0.001) with child mortality (Table 2). The coefficient for the corruption perception index [β = −0.2482; 95% C.I: −0.2482; −0.1906] in the univariate model would mean that an increase in 1 point of the CPI would decrease the logarithm transformation of children deaths per 1000 live births by 0.2482.

**Table 2:** Univariate and multivariate linear regressions predicting Log of children deaths per 1000 live births in 178 countries, 2008 (£).

	Univariate analysis				Multivariate analysis			
	Estimate	Std Error	t value	Pr(>|t|)	Estimate	Std Error	t value	Pr(>|t|)
Intercept	-	-	-	-	1.7925	0.2242	7.997	**<0.001**
**Socio economic factors**								
Log10(GDP per capita)	−0.7486	0.0348	−21.490	**<0.001**	-	-	-	-
People with access to improved water source	−0.0404	0.0036	−11.290	**<0.001**	-	-	-	-
People with access to improved sanitation	−0.0169	0.0009	−19.590	**<0.001**	−0.0050	0.0010	−4.786	**<0.001**
Percentage of rural population	0.0166	0.0016	10.703	**<0.001**	-	-	-	-
Literacy rate	−0.0243	0.0017	−14.180	**<0.001**	-	-	-	-
Dependency ratio	0.0637	0.0054	11.800	**<0.001**	0.0096	0.0016	6.162	**<0.001**
Density	−0.0002	0.0001	−2.173	**0.0311**	-	-	-	-
**Health and medical factors**								
Log10(Total health expenditure per capita)	−0.7075	0.0309	−22.920	**<0.001**	−0.1714	0.0619	−2.769	**0.006**
Health expenditure (% GDP)	−0.0723	0.0167	−4.323	**<0.001**	-	-	-	-
DTP coverage (%)	−0.0233	0.0025	−9.207	**<0.001**	−0.0027	0.0016	−1.720	0.0877
Measles coverage (%)	−0.0242	0.0024	−10.000	**<0.001**	-	-	-	-
Food supply	−0.0009	0·0001	−16.070	**<0.001**	-	-	-	-
**Environmental factors**								
Presence of an equatorial climate on the national territory	0.5572	0.0802	6.949	**<0.001**	-	-	-	-
Presence of an arid climate on the national territory	0.4718	0.0838	5.634	**<0.001**	0.1012	0.0366	2.763	**0.006**
Presence of a warm temperate climate on the national territory	−0.5316	0.0815	−6.524	**<0.001**	−0.0749	0.0388	−1.928	0.0558
Presence of a polar or snow climate on the national territory	−0.4359	0.0966	−4.514	**<0.001**	-	-	-	-
Log10(Percentage of national population affected by flood)	0.0892	0.0213	4.178	**<0.001**	-	-	-	-
**Political and societal factors**								
Civil liberties	0.1880	0.0210	8.959	**<0.001**	-	-	-	-
Political rights	0.1404	0.0185	7.596	**<0.001**	-	-	-	-
Corruption perception index	−0.2194	0.0147	−14.980	**<0.001**	−0.0644	0.0158	−4.088	**<0.001**
Log10(National battle-related deaths/person)	0.0802	0.0433	1.853	0.0656	-	-	-	-

£: Analysis of deaths in children from a multivariate linear regression model. Only variables with a p.value<0·1 in univariate regression were incorporated in the final multivariate analysis. Backward stepwise procedure with a stop criterion fixed at 0·1 was then applied to determinate the final multivariate model.

For child mortality rates, the final multivariable model (adjusted R^2^ = 0.890) included the following significant variables: percentage of people with improved sanitation (p.value<0.001), logarithm of total health expenditure (p.value = 0.006), CPI score (p.value<0.001), presence of an arid climate on the national territory (p = 0.006), and the dependency ratio (p.value<0.001). In this model, presence of a warm temperate climate on the national territory and DTP vaccination coverage were also found as almost significant (p<0.1). Incorporation of the CPI in the model increased the adjusted R^2^ from 0.8736 to 0.8897 (+1.61%). The coefficient for the corruption perception index [β = −0.0644; 95% C.I: −0.0954; −0.0334] in the final multivariate model would mean that a decrease in 1 point of the CPI score would increase the log of child deaths per 1000 live birth by 0.0644.

Plot of residuals versus predicted values and qqplot did not revealed particular pattern and indicated that homoscedasticity and normal hypothesis were respected in the final linear model. Variance inflation factors of variables included in the final model were also found to be lower than 5 showing that the magnitude of multicollinearity was low.

## Discussions

Ecological studies have their limitations (i.e. bias, confounding) [33]. The available data in a country may not have been sufficient to reflect the local variations of all the studied variables. This study was limited to a one year period (2008) and, due to the lack of data, some important variables to explain child mortality (as breastfeeding for example [34]) could not be included in the present analysis. Furthermore, several variables were based on estimations made by the United Nations and their quality could vary greatly compared to other objective data used in this analysis. However, all the data used in this analysis is the only available to study the problem at this scale. Although there are limitations, the fact that the present analysis came to similar observations on suspected and described phenomenon acting on child mortality rates is unlikely to have been due to a statistical artefact.

The linear regression analysis presented here, after taking into account known significant predictors of children deaths (mainly health expenditure, sanitation level, dependency ratio and arid climate) seems to point towards a significant pejorative link between perceived corruption and death rate at national level. In this model, a decrease in CPI of one point (i.e. a more important perceived corruption) was associated with an increase in log of national child mortality rate of 0.0644. The adjustment of the final model was very good (Pseudo R^2^ = 0.89) meaning that 89% of the data variability was captured by modelisation. Incorporation of CPI in the final model made a rise in the Pseudo R^2^ of 1.61%. According to this result, it could be hypothesized that roughly 1.6% of world deaths in children could be explained by corruption meaning that, of the annual 8.795 million children deaths, more than 140000 annual children deaths could be indirectly attributed to corruption.

By its simplistic nature, the model constructed here, has probably a non negligible tendency to underestimate the effects of corruption on children deaths. As shown elsewhere, across different country contexts, corruption has been a cause and consequence of poverty and so could possibly directly impacts the others significant parameters retrieved by the analysis, especially the percentage of population with adequate access to sanitation [35] and the public health expenditure [36]. Growing evidence from around the world indicates that corruption, fraud, and abuse are also resulting in significant losses of public money and denial of good quality health and sanitation services to millions of people [1], [2], [4], [7]. The diversity of health and water/sanitation systems worldwide, the multiplicity and complexity of parties involved make it difficult to determine the overall impacts of corruption in children deaths around the globe. Local studies have to be made to elucidate the underground complexity of the relation between child health, socio-economic conditions, and corruption.

This study is a first step in the quantification of the current global impact of corruption on child deaths in human populations and shows that annually at least 140000 children deaths could be related to corruption, a total that largely exceeds the conspicuous pooled total of cholera, rabies, Ebola and combat-related deaths. But still, because the equation corruption = deaths is seldom explicit, corruption only seems like a nuisance.

The global response to child deaths must involve a necessary increase in funds available to 1) develop water and sanitation access and 2) purchase new methods for prevention, management, and treatment of major diseases killing children around the globe (principally pneumonia, diarrhoea and malaria). However, without paying attention to the anti-corruption mechanisms needed to ensure their proper use, it will also provide further opportunity for corruption. In practice, donors and governments still treat health, water/sanitation access and corruption as separate rather than integral components of the same strategy. To address these obstacles, designing dedicated indicators at micro and macro levels which monitor efficiently corruption impacts on health and heath related services, is urgently needed. Policies and interventions supported by governments and donors must integrate initiatives that recognise how health and corruption are inter-related.
